# Analysis of the Consequences of the COVID-19 Pandemic on People with Severe Mental Disorders

**DOI:** 10.3390/ijerph18168549

**Published:** 2021-08-13

**Authors:** Antonio José Sánchez-Guarnido, Nuria Hidalgo, Jorge Arenas de la Cruz, Inmaculada Esteban, Silvia Mondón, Carlos Herruzo

**Affiliations:** 1 Hospital Santa Ana, 18600 Motril, Spain; antonioj.sanchez.guarnido.sspa@juntadeandalucia.es (A.J.S.-G.); nuriahidalgo96@gmail.com (N.H.); inmaestebanpi@gmail.com (I.E.); 2 Hospital General Universitario Ntra. Sra. del Prado, 45600 Talavera de la Reina, Spain; jarenasd@sescam.jccm.es; 3 Hospital Clínic de Barcelona, 08036 Barcelona, Spain; smondon@clinic.cat; 4 Department of Psychology, Universidad de Córdoba, 14071 Córdoba, Spain

**Keywords:** severe mental disorder, adherence to preventive guidelines against COVID-19, psychotherapy, occupational therapy, nursing

## Abstract

For people with severe mental disorders (SMDs) the COVID-19 pandemic may pose a number of risks. These include the loss of needed care, a higher probability of infection, and the worsening of their mental health. To analyze the pandemic’s impact on care received, relapses, loss of employment, and adherence to preventive guidelines in SMD sufferers, a multicenter retrospective cohort study was carried out comparing 185 patients diagnosed with SMD and 85 with common disorders. The results showed that during lockdown, there was a significant reduction in face-to-face psychotherapeutic, nursing, and occupational therapy interventions. In the same period, telematic interventions were introduced which, although subsequently reduced, now continue to be used to a greater extent than before the pandemic. Employment decreased significantly (13% vs. 9.2%; 𝜒^2^ = 126.228 *p* < 0.001). The percentage of people with SMD following preventive guidelines was significantly lower for both hand washing (56.2% vs. 75.3%; 𝜒^2^ = 9.360, *p* = 0.002) and social distancing (47% vs. 63.5; 𝜒^2^ = 6.423 *p* = 0.011). In conclusion, the COVID-19 pandemic has led to a reduction in the interventions that are needed for the recovery of people with SMDs, together with a significant loss of employment and an increased risk of contagion due to less adherence to preventive guidelines. In the future, appropriate attention to these people’s needs must be guaranteed.

## 1. Introduction

The scientific literature has recorded how different pandemics have affected the mental health of the world’s population [1], but countries have nevertheless been caught unprepared to manage such repercussions at this level [2] and the COVID-19 pandemic has posed a great challenge for effective SMD therapy [3]. A recent meta-analysis has provided concrete figures on the prevalence of mental disorders during the pandemic. The study indicates that the prevalence of depression in COVID-19-affected populations is more than three times higher (15.97%) than in the general population (4.4%); while it is four times higher for anxiety (15.15% vs. 3.6%); and five times higher for post-traumatic stress disorder (21.94% vs. 4%) [4]. Along the same lines, another study has noted that, in patients with no previous psychiatric history, a diagnosis of COVID-19 was associated with a higher incidence of a first psychiatric diagnosis in the following 14 to 90 days [5]. Similarly, Hu et al. [6] found a positive relationship between the incidence of schizophrenia and the epidemic situation in China.

There is evidence that the continuity of care by mental health services is essential to avoid relapses [1]. At the same time, however, during the pandemic, face-to-face interventions also increased the risk of infection in patients and professionals, leading to a major change in the way care is provided. In October 2020, the WHO reported a drop of nearly 40% in both psychotherapy and psychosocial interventions in Europe since the onset of the crisis. This represents a challenge for programs providing services to patients with severe mental disorders (SMDs) [3]. In an attempt to alleviate the impact of this disconnection as much as possible, telephone and videoconference consultations were implemented as an alternative to face-to-face care. The use of telematic interventions, however, has raised a series of fears among mental health professionals regarding such issues as the loss of quality in the therapeutic relationship, the violation of confidentiality and privacy, insecurity regarding information received and certain legal regulations affecting clinical practice [7,8]. Specific training for health professionals in skills for this mode of therapy is also required [7,8], and full access by patients to these forms of treatment, in terms both of material resources and the skills for their effective use [8]. Less research has been carried out examining the effectiveness of telepsychiatry for people with SMDs, and we know that the use of digital technologies is lower in people with psychosis than in the general population [9]. Nevertheless, the implementation of modalities that include telephones, the internet, and videoconferencing appears to be feasible, in addition to the use of text messaging or social networking applications. Preliminary evidence suggests that these modalities contribute to patient improvement, although more data are needed [10]. It is also useful to analyze the limitations of existing research and the risks of using this technology [11].

Changes in the interventions received and other aspects related to COVID-19 can affect people with SMD at different levels. Some studies have related the appearance of psychotic symptoms with exposure to other coronaviruses [12] and other psychopathological alterations in people with SARS-CoV [13]. The association between influenza infection and psychosis has been reported since the Spanish influenza pandemic in the 20th century, and subsequent acute “influenza psychoses” have been documented during multiple pandemics [14]. It has also been observed that patients with COVID-19 but without psychiatric histories may develop psychotic symptoms [15]. Social distancing, by which governments attempt to decrease community transmission of COVID-19, may also have negative effects on mental health [16]. Research in China shows that fears of contracting COVID-19, together with social isolation, have had a moderate or severe psychological impact on more than half of the population (53.8%) [17]. If the stress associated with the pandemic worsens mental health in the general population, it is to be expected that its impact will be even greater in people with a previous SMDs [18]. We know that this group has experienced a greater increase in anxiety compared to the population without a psychopathological diagnosis [19], that social support improves community integration for people with schizophrenia and other psychotic disorders [20], and that the interruption of these social contacts puts patients’ recovery at risk [21]. Along the same lines, this group may also see the coverage of their basic needs diminished, given that many of these patients are heavily dependent on community support services (employment, occupational, mutual support, etc.) which are difficult to access in these circumstances [22]. Unemployment and economic problems, which have increased significantly during the COVID-19 pandemic, may also be particularly associated with SMD sufferers [21,22,23,24].

In addition to the effects of the pandemic on mental health, it is also relevant to know the risk of contagion. A significantly higher risk of infection has been observed in patients diagnosed with mental disorders [3]. Thus, one study found that the odds of being diagnosed with COVID-19 were higher for patients with attention deficit hyperactivity disorder, bipolar disorder, depression, and schizophrenia [16]. In addition, having a psychotic spectrum disorder is an important risk factor for COVID-19 mortality [25], as people with SMDs are at increased risk for greater physical complications than the general population [26]. In relation to this increased vulnerability for COVID infection in people with SMDs, different authors have established a relationship between SMDs and a lower adherence to protective measures [13,27,28]. Thus, several studies evidence a greater difficulty in the implementation of preventive measures by people with SMDs [29]. In patients with severe psychopathological symptoms, difficulties may appear in the understanding of preventive guidelines, as well as greater interference in the skills to carry them out [30].

In summary, the efforts invested in the investigation of COVID-19 and its physical consequences should not obviate the need to focus on the illness’s psychosocial repercussions, especially in vulnerable groups such as people with SMDs. Spain, where this study was carried out, has been one of the countries with the highest incidence of cases and the strictest initial lockdown, but even so there are remarkably few published studies addressing the consequences of the COVID-19 pandemic in Spanish SMD patients. Our objective was therefore to study the pandemic’s impact on this population group, including variables such as changes in the care received, consequences in terms of relapses, social variables such as employment, and the following of preventive guidelines.

## 2. Materials and Methods

### 2.1. Design

Multicenter retrospective cohort study.

### 2.2. Participants

In order to compare the consequences of the pandemic in people with SMDs in relation to patients with other mental disorders, it was decided to select a group of SMD patients whose inclusion criterion was a diagnosis of schizophrenia or other psychoses, bipolar disorder, or personality disorder, with a group of patients with other diagnoses. We chose to carry out the study in day hospitals because we consider them to be facilities where patients from both groups are usually found and where the clinical histories are very complete and all the data necessary to carry out a good retrospective study can be found.

Thus, the study included patients under follow-up in mental health day hospitals (MHDHs) at some point in the year 2020. Fifteen hospitals of the Spanish National Health System participated in the study and a sample of 185 people diagnosed with SMDs and 85 diagnosed with common disorders were included. The diagnoses of the SMD group included schizophrenia or other psychotic disorders (44.3%), personality disorder (40.5%), and bipolar disorder (15.1%); those of the control group included depressive disorders (30.6%), anxiety disorders (16.5%), substance use disorders (21.2%), eating disorders (5.9%) and others (25.9%). The mean age was statistically lower (*t* = 3.374; *p* < 0.001) in the SMD group (38.29) than in the control group (43.21). There were differences in terms of household composition (𝜒^2^ = 18.239; *p* < 0.006), with a lower percentage of patients in the SMD group having their own family home (21.6% vs. 44.7%). In terms of employment, although the difference was not statistically significant (*p* < 0.138), there was a lower percentage of employed people in the SMD group (13.0% vs. 24.7%) prior to the onset of the pandemic. There were no significant differences in sex or levels of education (see Table 1).

### 2.3. Procedure

Data were collected retrospectively. The collaborating clinicians from each institution reviewed the medical records and during the months of October and November 2020 conducted an interview with the patients. The interview focused on adherence to preventive guidelines, and they were asked how often they followed the recommendation of mask use, hand washing, and maintaining social distance. Patients responded on a scale of 1 (never) to 5 (always). The rest of the variables were collected from the patient’s clinical history. Prior to participation in the study, each patient was informed both orally and in writing of the objectives of the project and its methodology, and informed consent was obtained using the corresponding form. A database was designed to which only the investigators had access and the clinical data were processed without any patient-identifying information.

### 2.4. Variables

The sociodemographic variables studied were age, sex, household composition, employment status, and educational level.

Two exposure variables were analyzed. The first was defined by the presence of an SMD diagnosis (schizophrenia or other psychotic disorders, bipolar disorder, or personality disorder) as opposed to a common mental disorder (depressive disorder, anxiety disorders, or other diagnoses). In relation to this exposure, we studied whether the patient always followed the recommended prevention guidelines. Here, three variables were included: the use of masks, hand washing, and social distancing. They were collected through patient interviews and categorized dichotomously. The preventive guideline was considered to be met when the patient responded that he/she always performed the behavior.

The second exposure variable was the time period in relation to the pandemic and lockdown. Three two-month observation periods were established: pre-lockdown period (16 January to 15 March 2020), lockdown period (16 March to 15 May 2020), and post-lockdown period (16 May to 15 July 2020).

The response variables associated with this second exposure variable were related to changes in the interventions received and to indicators of how the pandemic affected the mental health of these patients. All these variables were collected from the patients’ clinical histories. The psychotherapy, occupational therapy, and nursing interventions received in person and telematically in each period were coded dichotomously.

As indicators of how the pandemic affected the mental health of patients, data were collected for admission to inpatient units (dichotomous), emergency consultations (mean number of emergencies per patient), and employment (dichotomous: employed/unemployed).

### 2.5. Data Analysis

Statistical analysis of the data was performed using the IBM-SPSS V.21.0 program. The results of the categorical variables were expressed as percentages and those of the quantitative variables as mean and standard deviation. The chi-square test was used to compare proportions between groups and a Student’s *t*-test for independent samples was used to analyze the differences between the mean values of the quantitative variables.

To analyze whether there were significant changes between the measurement of a variable at different times in the study, a Student’s *t*-test for repeated samples was used for quantitative variables and McNemar’s test was used for dichotomous variables. The level of statistical significance used was *p* < 0.05.

## 3. Results

### 3.1. Changes in Care Received

We began by comparing the percentages of patients with SMDs who received face-to-face or telemedicine psychotherapy before, during, and after lockdown (see Table 2). The percentage of patients who received face-to-face psychotherapy went from 81% in the pre-pandemic period to 25.9% during lockdown, returning to 79.5% after lockdown. These differences are statistically significant between pre-lockdown and lockdown (𝜒^2^ = 91.080; *p* < 0.001), and between lockdown and post-lockdown (𝜒^2^ = 91.080; *p* = 0.000), but are not significant between pre-lockdown and post-lockdown (𝜒^2^ = 0.098; *p* = 0.755). Patients receiving psychotherapy telematically increased from 4.4% in the pre-lockdown period to 64.8% during lockdown, and to 30% after lockdown. These differences are statistically significant between pre-lockdown and lockdown (𝜒^2^ = 159.055; *p* < 0.001), between lockdown and post-lockdown (𝜒^2^ = 73.297; *p* < 0.001), and between pre-lockdown and post- lockdown (𝜒^2^ = 61.653 *p* < 0.0010).

We then compared the percentages of patients with SMDs who received some type of occupational therapy intervention, either face-to-face or telematically. The percentage of patients who received face-to-face occupational therapy in the different periods went from 47% prior to lockdown to only 8.1% during lockdown, returning to 45.4% after lockdown. These differences are statistically significant between the pre-lockdown and lockdown period (𝜒^2^ = 61.476; *p* < 0.001) and between lockdown and post-lockdown (𝜒^2^ = 63.342; *p* < 0.001), but are not significant between pre-lockdown and post-lockdown (𝜒^2^ = 0.108; *p* = 0.742). Patients receiving occupational therapy telematically went from 2.2% in the pre-lockdown period to 38.1% during lockdown, and to 22.6% after lockdown. These differences are statistically significant between pre-lockdown and lockdown (𝜒^2^ = 95.010; *p* < 0.001), between lockdown and post-lockdown (𝜒^2^ = 27.113; *p* < 0.001), and between pre- lockdown and post-lockdown (𝜒^2^ = 49.424; *p* < 0.001). Like psychotherapy, telematic health interventions in occupational therapy were also introduced during lockdown and, although they were subsequently reduced, they are now still used to a greater extent than before lockdown.

Finally, the percentages of patients who received some type of nursing intervention were analyzed. The percentage of patients who received face-to-face nursing interventions in the different periods went from 61.1% before lockdown to 17.8% during lockdown, returning to 61.1% afterwards. There are significant differences between the pre-lockdown and lockdown periods (𝜒^2^ = 72.570; *p* < 0.001) and between lockdown and post-lockdown (𝜒^2^ = 78.013; *p* < 0.001), but the differences are not significant between the pre-lockdown and post-lockdown (𝜒^2^ = 0; *p* = 1) periods. The percentage of patients who received nursing interventions telematically increased from 4.4% in the pre-lockdown period to 39.6% during lockdown and 23.3% after lockdown. These differences are statistically significant between pre-lockdown and lockdown (𝜒^2^ = 91.093; *p* < 0.001), between lockdown and post-lockdown (𝜒^2^ = 24.329; *p* < 0.001), and between pre-lockdown and post-lockdown (𝜒^2^ = 40.984; *p* < 0.0010).

### 3.2. Impact of the Pandemic on Admissions, Emergencies, and Employment

The percentages of patients diagnosed with SMD who were admitted to inpatient units in the different periods are shown below in Table 2. Thirteen percent were admitted during the period prior to lockdown, dropping to 8.1% during lockdown and then rising to 10.8% after lockdown. These differences are not statistically significant between the pre- lockdown and lockdown periods (*p* = 0.108), between the pre-lockdown and de-escalation periods (*p* = 0.584), or between lockdown and post-lockdown (*p* = 0.405).

In relation to emergency consultations in the different periods, the mean number of emergencies per patient in our sample went from 0.27 in the period prior to lockdown to 0.41 during lockdown and 0.39 after lockdown. These differences are not statistically significant, between pre-lockdown and lockdown (*t* = −0.988, *p* = 0.324), between pre-lockdown and de-escalation (*t* = −0.558, *p* = 0.577), or between lockdown and post-lockdown (*t* = 0.222, *p* = 0.825).

Statistically significant differences were found in employment. Before the pandemic, 13% of the patients with SMDs in our sample had jobs, but this fell to 9.2% during the pandemic (𝜒^2^ = 126.228 *p* < 0.001).

### 3.3. Following of Preventive Guidelines

Table 1 also shows the following of preventive guidelines. The percentage of patients who always wore a mask was lower in the SMD group, although this is not statistically significant (73% vs. 82.4%; 𝜒^2^ = 2.918, *p* = 0.88). Bigger differences were found in the case of hand washing, with a significantly lower percentage of people always following the guidelines in the SMD group (56.2% vs. 75.3%; 𝜒^2^ = 9.360, *p* = 0.002), and in social distancing, where less adherence to the guidelines was also observed in the SMD group (47% vs. 63.5; 𝜒^2^ = 6.423 *p* = 0.011).

## 4. Discussion

Although the COVID-19 pandemic has had an impact on the entire population, there are certain special risk groups. This study focuses on one of these groups: people with SMDs. According to our results, the lockdown situation in the first wave of the pandemic and in the ensuing months affected several areas, including changes in the health care received by SMD patients, a significant loss of employment, and less following of preventive measures.

According to the World Health Organization [31], in 2020, 60% of countries classified mental health services as essential health services. Despite this, in Spain, some mental health services have been markedly reduced. In the health facilities participating in this study, it was found that a significant percentage of people (in some cases more than 50%) did not receive any psychotherapeutic or occupational therapy or nursing care during the period of lockdown. In the same vein, the data provided in the portals of the various regional health services to which the clinics in this study belong show that the waiting lists [32] for first appointments with mental health specialists (psychiatrists and clinical psychologists) underwent considerable variations, both in the number of people listed and in the number of days they had to wait for their first appointment. In the case of occupational therapy interventions, the data obtained in our study are very similar to those disseminated by the General Council of Colleges [33], which in May 2020 published that 66.37% of the occupational therapists surveyed estimated that during lockdown they had been able to replace face-to-face treatment with other means in less than 25% of cases. It is relevant that, assuming the special vulnerability of the people using these mental health resources, the level of interruption in treatment was of a large magnitude. It should be noted that in all three levels of care (psychotherapy, occupational therapy, and nursing) the level of face-to-face treatment at the end of lockdown had returned to the pre-lockdown level. The figures for the interruption of care during lockdown may be attributable to multiple reasons, one of these being the relocation of some professionals, especially nurses, to other care resources directly dedicated to people affected by coronavirus. By April 2020, for example, nearly one in five nurses nationwide had been transferred to another facility or service to treat COVID-19-positive patients [34].

On the other hand, the data collected confirm that telematic care by video call at those three levels was non-existent prior to lockdown. Psychotherapeutic, occupational therapy, and non-face-to-face nursing interventions prior to lockdown accounted for around 5% of the total and were carried out entirely by telephone. Considering this starting situation, it was inevitably difficult, in the midst of a health emergency, to optimally articulate an alternative service to compensate for the lack of face-to-face care. Although alternative non-face-to-face means were provided to continue with therapeutic programs, those based on the use of videoconferencing were not sufficient. Psychotherapy, occupational therapy, and nursing were unavailable for more than ten percent of the patients. To explain this, we must also consider the possible effect of the lack of technological prerequisites, such as equipment and its use by a significant number of patients and professionals. For professionals and patients, the lockdown situation represented a first experience with electronic mental health, in often suboptimal circumstances, with therapists and patients experiencing difficulties in finding adequate resources or private spaces for virtual communication to enable bonding. Some of these difficulties had already been reported in studies prior to the pandemic [7,8,35]. However, the evidence is generally favorable in terms of acceptance and satisfaction by patients and professionals [36], feasibility, and efficacy, without a negative effect on the therapeutic relationship [7,8,37]. The evidence is favorable, therefore, regarding the effectiveness of these pathways from different therapeutic models and in users with various mental disorders [38,39,40].

Regarding the consequences of the pandemic in terms of admissions, emergencies, and employment, as compared to the pre-pandemic phase, the percentage of patients admitted was lower during lockdown and then recovered in the post lockdown phase, but without any statistically significant differences. As for emergency consultations, the patients in our sample had more visits to emergency departments during lockdown and post-lockdown compared to the previous period, although, again, these differences were not statistically significant. Little previous research exists on this subject, but some authors point out that patients with pre-existing mental disorders may have a higher risk of relapse or new episodes due to the stress associated with the COVID-19 pandemic [17,41]. It has also been observed that psychiatric symptoms worsened in patients with pre-existing psychopathological disorders [42,43]. In light of these earlier studies, we cannot conclude that the results obtained in our study necessarily imply that there was no increase in relapses, and different factors may have mediated between relapse and the circumstances of each patient’s hospital admission or visits to the emergency department. One thing that may have had an influence is changes to the criteria for access to hospitals. The Chinese Society of Psychiatry suggested, for example, that psychiatric hospitals should tighten admission criteria [44]. Additionally, something similar also occurred in Spain, where the criteria for hospital admission became more restrictive [45,46,47,48]. The paralysis of activity during the period of lockdown also had an impact on the transportation and distribution of medical resources worldwide, and these changes in infrastructure and mobility could have contributed to reducing access to mental health services [43].

On the other hand, and in order to consider the overall impact of the situation generated by COVID-19, our study also included the pandemic’s repercussion on employment. According to our data, the percentage of employed people with SMDs decreased significantly during the pandemic, with employment levels already being very low among this group. Suffering from a severe mental disorder limits access to employment, and reversing this must be one of the priority care objectives right from the start [49], especially considering its benefits in terms of economic independence and self-identity, the continuance of physical and mental activity, the structuring of time, and the generation and strengthening of interpersonal relationships and communication. In short, employment, together with other social and economic variables, and its interaction with other psychological variables (social skills, social anxiety, etc.), is a key issue in understanding the recovery of people with SMDs, and one of the great challenges facing our society with respect to this group. Studies available in this field are remarkably scarce [41], so further analysis of this variable could be an interesting subject of future research.

Regarding preventive strategies, we found a significantly lower percentage of patients who always washed their hands and observed social distancing rules. Adherence to preventive guidelines may be undermined by worsening psychopathology together with other social and economic variables, and its interaction with other psychological variables (social skills, social anxiety, etc.) [50]. Several studies link poor adherence to preventive measures with greater difficulty in understanding and following instructions among SMD sufferers [51]. The family and social support received by the patient may also be important and strategies are therefore recommended to compensate for the obstacles these people face, with emphasis on the modeling of hand hygiene and disinfection guidelines, the use of masks, and social distancing [30]. The Italian Psychiatric Association has published a series of guides with operational instructions on prevention and health that could be adopted by mental health departments [52].

The present study focuses on people with SMDs, a group vulnerable to the COVID-19 pandemic, and with a greater need for research. By using a longitudinal approach, albeit retrospectively, it was possible to observe changes over time in relation to the interventions carried out with these patients. The study also included a control group with less severe patients, to compare the use of preventive measures. The multicenter nature of the study provided a larger sample and greater potential for generalization of the results. The use of day hospitals as participating institutions ensured greater reliability in data collection, with all the hospitals having very reliable patient records.

However, the study also had some limitations. At the methodological level, conducting a retrospective study always involves a greater risk of bias, and a larger sample would have allowed us to check whether some of the differences observed in our study had become significant. It would also have been interesting to have more accurate measurements of the interventions performed and their frequency, and of the impact of other variables at the psychopathological and social functioning levels. We therefore believe that it would be interesting in future research to carry out prospective studies, to add control groups without psychopathologies, and to use other means of measuring patients’ interventions and the changes in their recovery patterns.

## 5. Conclusions

The COVID-19 pandemic has entailed a series of risks for people with SMDs, starting with a reduction in the interventions necessary for their recovery, but also at a social and occupational level, the most dramatic example of which would be the loss of employment in this group, which in any case already suffers from high unemployment rates. This population group is also at special risk of contracting COVID-19 due to poorer adherence to preventive guidelines. For all these reasons, it is important for us to learn as a society, so that in future situations, such as those experienced in this pandemic, people with SMDs are not left unprotected by health and social institutions, and appropriate attention is ensured to meet both their physical and their mental health needs.

## Figures and Tables

**Table 1:** Sociodemographic and clinical variables in the sample.

Sociodemographics				
Variables	Total	Non-SMD Patients	SMD Patients	*p*
Age; mean (SD)	39.90 (11.81)	38.29 (11.60)	43.41 (11.59)	*t* = 3.374 <0.001
Gender				𝜒^2^ = 0.964 <0.617
Women	149 (55.2%)	48 (56.5%)	101 (54.6%)	
Men	119 (44.1%)	37 (43.5 %)	82 (44.3%)	
Non-binary gender	2 (0.7%)	0 (0.0%)	2 (1.1%)	
Total	270 (100%)	85 (100%)	185 (100%)	
Household composition				𝜒^2^ = 18.239 <0.006
Full family of origin (parents with or without siblings)	78 (28.9%)	18 (21.2%)	60 (32.4%)	
Own family household (married, cohabiting, and/or with children)	78 (28.9%)	38 (44.7%)	40 (21.6%)	
Horizontal (with friends or siblings)	16 (5.9%)	2 (2.4%)	14 (7.6%)	
Single parent (single parent with or without siblings)	37 (13.7%)	9 (10.6%)	28 (15.1%)	
Other	7 (2.6%)	2 (2.4%)	5 (2.7%)	
Single person	46 (17%)	15 (17.6%)	31 (16.8%)	
Institutionally supervised housing (sheltered housing, group home, etc.)	8 (3%)	1 (1.2%)	7 (3.8%)	
Pre-pandemic				𝜒^2^ = 8.347 <0.138
Work/Vocational/Occupational Activity				
Students	20 (7.4%)	4 (4.7%)	16 (8.6%)	
TIW	54 (20%)	19 (22.4%)	35 (18.9%)	
Retired, pensioners	79 (29.3%)	20 (23.5%)	59 (31.9%)	
Unemployed	71 (26.3%)	21 (24.7%)	50 (27%)	
Working	45 (16.7%)	21 (24.7%)	24 (13%)	
Volunteer/mutual aid agents	1 (0.4%)	0 (0%)	1 (0.5%)	
Level of Education				𝜒^2^ = 7.137 <0.129
No education	8 (3%)	1 (1.2%)	7 (3.8%)	
Primary (E.G.B., ESO)	96 (35.6%)	32 (37.6%)	64 (34.6%)	
Secondary (B.U.P., baccalaureate, vocational training)	112 (41.5%)	30 (35.3%)	82 (44.3%)	
University graduate (bachelor’s degree)	40 (14.8%)	14 (16.5%)	26 (14.1%)	
Post-graduate (graduate, master’s, doctorate)	14 (5.2%)	8 (9.4%)	6 (3.2%)	
**Clinics**				
Following of prevention guidelines				
Use of masks at all times	205 (75.9%)	70 (82.4%)	135 (73%)	𝜒^2^ = 2.803 <0.094
Hand washing at all times	168 (62.2%)	64 (75.3%)	104 (56.2%)	𝜒^2^ = 9.018 <0.003
Social distancing at all times	141 (52.2%)	54 (63.5%)	87 (47%)	𝜒^2^ = 6.357 <0.012

SD: Standard Deviation; G.B.E.: General Basic General Education; ESO: Compulsory Secondary Education; TIW: Temporary Inability to Work.

**Table 2:** Mental health consequences of the pandemic and changes in care received.

Clinics						
Variables	% Pre-Lockdown Patients (16 January–15 March)	% Lockdown Patients (16 March–15 May)	% Post-Lockdown Patients (16 May–15 July)	Before and during Lockdown *p*	Pre- and Post-Lockdown *p*	During and Post-Lockdown *p*
Changes in care received						
Face-to-face psychotherapy	81.1	25.9	79.5	𝜒^2^ = 91.080 0.001	𝜒^2^ = 0.098 0.755	𝜒^2^ = 91. 467 0.001
Telematic psychotherapy	4.4	64.8	30	𝜒^2^ = 159.055 0.001	𝜒^2^ = 61.653 0.001	𝜒^2^ = 73.297 0.001
Changes in care received						
Face-to-face occupational therapy	47	8.1	45.4	𝜒^2^ = 61.476 0.001	𝜒^2^ = 0.108 0.742	𝜒^2^ = 63.342 0.001
Telematic occupational therapy	2.2	38.1	22.6	𝜒^2^ = 95.010 0.001	𝜒^2^ = 49.424 0.001	𝜒^2^ = 27.113 0.001
Changes in care received						
On-site nursing	61.1	17.8	61.1	𝜒^2^ = 72.570 0.001	𝜒^2^ = 0.000 1.000	𝜒^2^ = 78.013 0.001
Telematic nursing	4.4	39.6	23.3	𝜒^2^ = 91.093 0.001	𝜒^2^ = 40.984 0.001	𝜒^2^ = 24.329 0.001
Urgent consultations (means)	0.27	0.41	0.39	*t* = −0.988 0.324	*t* = −0.558 0.577	*t* = 0.222 0.825
Admissions to the hospitalization unit	13	8.1	10.8	0.108	0.584	0.405
Changes in employment	13		9.2	𝜒^2^ = 126.228 0.001		

## Data Availability

The data presented in this study are available on request from the corresponding author. The data are not publicly available because they are part of an ongoing project.
